# Active training for amblyopia in adult rodents

**DOI:** 10.3389/fnbeh.2015.00281

**Published:** 2015-10-27

**Authors:** Alessandro Sale, Nicoletta Berardi

**Affiliations:** ^1^Neuroscience Institute, National Research CouncilPisa, Italy; ^2^Department of Neuroscience, Psychology, Drug Research and Child Health NEUROFARBA, University of FlorenceFlorence, Italy

**Keywords:** amblyopia, environmental enrichment, physical exercise, visual perceptual learning, GABAergic inhibition

## Abstract

Amblyopia is the most diffused form of visual function impairment affecting one eye, with a prevalence of 1–5% in the total world population. Amblyopia is usually caused by an early functional imbalance between the two eyes, deriving from anisometropia, strabismus, or congenital cataract, leading to severe deficits in visual acuity, contrast sensitivity and stereopsis. While amblyopia can be efficiently treated in children, it becomes irreversible in adults, as a result of a dramatic decline in visual cortex plasticity which occurs at the end of the critical period (CP) in the primary visual cortex. Notwithstanding this widely accepted dogma, recent evidence in animal models and in human patients have started to challenge this view, revealing a previously unsuspected possibility to enhance plasticity in the adult visual system and to achieve substantial visual function recovery. Among the new proposed intervention strategies, non invasive procedures based on environmental enrichment, physical exercise or visual perceptual learning (vPL) appear particularly promising in terms of future applicability in the clinical setting. In this survey, we will review recent literature concerning the application of these behavioral intervention strategies to the treatment of amblyopia, with a focus on possible underlying molecular and cellular mechanisms.

## Introduction

During development, brain plasticity is high and neurons adapt promptly in response to environmental stimuli, but with the passage from youth to adulthood neural circuits become much less plastic. The decay in plasticity levels strongly prevents, in the mature brain, the possibility for functional recovery from developmental disorders. This condition is epitomized by amblyopia, a visual disorder affecting thousands of people; if not precociously recognized and treated, amblyopia has proven to be substantially insensitive to treatment after the age of 7–8 years. However, recent research has demonstrated the previously unsuspected possibility to elicit robust plasticity in the adult visual system and to promote recovery of visual abilities even in adult amblyopic subjects.

In the present survey, we shall review the recent literature on this hot topic, focusing on two non invasive treatment strategies which hold promise for successful clinical application in amblyopes: physical exercise and visual perceptual learning (vPL). In the first section, we introduce the relation between amblyopia and critical period (CP) for experience-dependent plasticity in the visual cortex, both in humans and in animal models. In the second section, we review the experimental strategies showing the possibility for recovery from amblyopia, starting with the description of the striking results obtained with pharmacological approaches acting on visual cortical plasticity and following with the discussion of the effects elicited by environmental enrichment procedures based on an increase in sensory-motor stimulation. In the third section, we shall focus on recent data showing that physical exercise and vPL are behavioral interventions particularly suited for cortical plasticity enhancement and recovery from amblyopia. In the last section, we discuss possible cellular and molecular mechanisms underlying the beneficial effects of physical exercise and vPL on visual cortical plasticity. Finally, we underline possible open questions and future research directions.

## Amblyopia and its Modeling in Laboratory Animals

Amblyopia (lazy eye), is a major developmental visual disorder that occurs in 1–5% of the world population and is typically caused by an early abnormal visual experience which occurs during a well defined CP, which is around 6–8 years of age in children. Typical causes of amblyopia are strabismus, image degradation due to refractive errors, or congenital cataract (Holmes and Clarke, 2006). The most common form of the disorder, unilateral amblyopia, results in a marked visual acuity impairment in the affected eye, together with reduced stereopsis, and low contrast and motion sensitivity. The physiology of the retina is generally spared in amblyopia (e.g., Sherman and Stone, 1973; Kratz et al., 1979; Baro et al., 1990), while the lateral geniculate nucleus of the thalamus (LGN) can appear atrophic (Wiesel and Hubel, 1963). There is large consensus, however, that amblyopia is prevalently caused by neural dysfunctions occurring in the primary visual cortex (V1; see Hess, 2001; Barrett et al., 2004).

Most important, recovery of normal visual functions is almost impossible after CP end, i.e., after 8 years of age in children. Thus, the CP for the appearance of amblyopia is paralleled by a sensitive period for the success of therapeutic strategies (see Lewis and Maurer, 2009).

Use of animal models of amblyopia has largely increased our knowledge on the neural mechanisms underlying this pathology. In kittens and rodents, the most widely diffused model of amblyopia is obtained by strongly reducing visual input to one eye by lid suture, a procedure usually referred to as monocular deprivation (MD). MD performed during the CP decreases the presence of binocular neurons in V1, reduces the number of neurons responding more vigorously to the deprived than to the non deprived eye, resulting in an increase of neurons dominated by the open eye; behaviorally, a loss of binocular vision and a strong reduction of visual acuity and contrast sensitivity for the deprived eye is observed (e.g., Wiesel and Hubel, 1963; Hubel and Wiesel, 1970; Olson and Freeman, 1975; Movshon and Dürsteler, 1977; Pizzorusso et al., 2006; Sale et al., 2007).

As found in humans, the effects of MD can be at least partially reversed if normal visual input is reestablished during the CP (e.g., Blakemore et al., 1981; Antonini and Stryker, 1998; Pizzorusso et al., 2006; Sale et al., 2007). The basic strategy for treating amblyopia during CP is to remove the defects preventing a clear retinal image and to promote strengthening of neural connections coming from the lazy eye. So called “passive methods” such as refractive correction, alone or in combination with fellow eye patching or atropine penalization, are widely employed, with a percentage of success correlating with the total number of treatment hours (Foley-Nolan et al., 1997; Simons et al., 1997; Pediatric Eye Disease Investigator Group, 2002; Wu and Hunter, 2006).

These strategies do not lead to vision recovery if applied well after CP end and no successful therapy has been available for adult patients, so that amblyopia in adult subjects has long been considered an irreversible condition; lack of recovery was attributed to the dramatic decline in visual cortex plasticity that accompanies the transition from youth to adulthood and prevents visual cortical connections from remodeling in favor of the deprived eye input, following removal of its visual defects (e.g., Blakemore et al., 1981; Antonini and Stryker, 1998; Pizzorusso et al., 2006; Sale et al., 2007).

## Challenging the Dogma of Irreversibility

If the lack of recovery from amblyopia in adult subjects is due to the low levels of plasticity of adult V1, enhancing V1 plasticity might allow the deprived eye input to regain access to visual cortical neurons, promoting recovery from amblyopia. Following this hypothesis, a new era of exciting experimental studies has recently started, leading to the demonstration and now widely accepted notion that a high degree of residual cortical plasticity can be unmasked in the adult brain (see, for instance, the review by Bavelier et al., 2010).

### Pharmacological Studies

The first studies have exploited pharmacological treatments to either target factors that brake adult plasticity and/or to enhance levels of endogenous permissive molecules.

The excitatory/inhibitory (E/I) balance in V1 rapidly emerged as a crucial factor in controlling the opening and time course of CP plasticity (Hensch, 2005; Sugiyama et al., 2008); in particular, direct pharmacologically reduction of GABAergic transmission in the adult visual cortex has proven to be a suitable strategy for the restoration of plasticity processes (Hensch, 2005; Harauzov et al., 2010; Baroncelli et al., 2011). Interestingly, also transplantation of embryonic inhibitory neuronal precursors into the visual cortex of post-CP animals can induce a second window of plasticity after the end of the natural CP (Southwell et al., 2010). Whether these treatments are also capable to promote recovery from amblyopia is still unknown. A change in V1 E/I balance has also been achieved indirectly, pharmacologically targeting neuromodulators, such as serotonin, norepinephrine, and acetylcholine, which are known to be strongly involved in visual plasticity (Kasamatsu and Pettigrew, 1976; Bear and Singer, 1986; Kilgard and Merzenich, 1998; Bao et al., 2001; Goard and Dan, 2009) and which proved to impinge on E/I balance and to promote recovery from amblyopia (e.g., Maya Vetencourt et al., 2008; Bavelier et al., 2010; Morishita et al., 2010; Sale et al., 2014, for review) As an example, chronic delivery in the drinking water of the selective serotonine-reuptake inhibitor (SSRI) fluoxetine caused a marked decrease of GABAergic inhibition levels in V1 and this decrease was crucial for fluoxetine to enhance V1 experience-dependent plasticity and to promote visual function recovery in adult amblyopic rats following reopening of the initially deprived eye and closure of the normal eye (Maya Vetencourt et al., 2008).

Other successful pharmacological studies have targeted the adult brain extracellular milieu, making it more permissive for experience-dependent plasticity. Chondroitinase ABC, an enzyme responsible for the degradation of chondroitin sulfate proteoglycans (CSPGs) in the extracellular matrix, enhances V1 experience-dependent plasticity and induces full recovery from amblyopia in adult rats (Pizzorusso et al., 2002, 2006). Interestingly, some of the effects elicited by Chondroitinase ABC could be mediated by modifications of intracortical inhibitory circuits occurring after degradation of extracellular matrix perineuronal nets (PNNs; Hensch, 2005). An involvement of intracortical inhibitory circuits and of CSPGs has also been shown for the effects on V1 plasticity of the orthodenticle homeobox 2 (Otx2) homeoprotein, which transfers to parvalbumin-positive GABAergic interneurons in the developing mouse visual cortex, acting as a direct trigger both for the opening and closure of the CP (Sugiyama et al., 2008). CSPGs are necessary to capture endogenous Otx2 at the surface of parvalbumin interneurons, via the so called RK peptide (Beurdeley et al., 2012). Reducing CSPGs by means of chondroitinase ABC leads to a reduction in the amount of endogenous Otx2 bound to parvalbumin cells, and so does infusion of RK peptide which competes with the endogenous one; both treatments lead to functional recovery in adult amblyopic mice (Beurdeley et al., 2012).

Finally, a very attractive and relatively new kind of pharmacological substances acts at the level of epigenetic modifications of the brain chromatin status (Zhang and Meaney, 2010). The closure of the CP in the mouse visual cortex has been linked to downregulation of histone H3 and H4 acetylation (Putignano et al., 2007); accordingly, infusion of histone deacetylase inhibitors enhances plasticity in adult V1, leading to functional recovery in amblyopic rats past the end of the CP (Silingardi et al., 2010).

### Non Pharmacological Studies

Results more suitable for clinical application have been obtained by means of non invasive treatments aimed at inducing an endogenous recapitulation of the brain states that enhance V1 plasticity. Dark exposure initiated in adulthood reactivates synaptic plasticity in the visual cortex, induces recovery of dendritic spine density of neurons and promotes vision rescue in long-term MD rats (He et al., 2007; Montey and Quinlan, 2011). Thus, these results raise the provocative concept that darkness might be a cure for vision loss in amblyopia, a fascinating approach which however, appears quite limited in terms of human application.

A more promising approach is based on somewhat opposite strategies leading to the optimization and enhancement of sensory stimuli. The progenitor of this kind of treatments is environmental enrichment (EE; van Praag et al., 2000; Sale et al., 2014), whereby laboratory rodents are reared in large social groups in wide and attractive cages where a variety of toys are available and changed frequently to stimulate motor activity, novelty and curiosity as determinants of exploratory behavior. Adult amblyopic rats reared under EE conditions display a full rescue of their visual functions (Sale et al., 2007): moreover, EE is also able to reopen the CP for V1 plasticity in response to MD, even in aged rats (Baroncelli et al., 2010; Scali et al., 2012). Importantly, exposure to EE does also result in a marked reinstatement of visual depth-perception abilities of adult amblyopic animals, as tested with the visual cliff task (Baroncelli et al., 2013).

Mediators of EE effects include E/I balance and neuromodulators as well as other well known determinant of CP plasticity such as brain-derived neurotrophic factor (BDNF) and CSPGs (see Sale et al., 2014). Neuromodulators were known to respond to EE since the very first studies in the 60s which reported an increase in acetylcholinesterase activity, with subsequent work confirming and extending this initial observation to the other neuromodulator systems, like the serotonin and noradrenaline systems (van Praag et al., 2000). In the visual cortex, an enhanced serotonin expression has been shown to be critical for plasticity enhancement in adult enriched rats (Baroncelli et al., 2010), a result linking the impact of EE on visual cortical plasticity to the previously discussed importance of neuromodulating systems for CP reopening in the mature visual cortex. Moreover, recovery of plasticity in enriched amblyopic animals is associated with reduction of GABA release in the visual cortex, as assessed by brain microdialysis, enhancement of BDNF expression and with decrease of CSPG condensation in perineuronal nets in the visual cortex (Sale et al., 2007).

## Active Training for Amblyopia

One step up toward the application of the EE paradigm to human subjects is to study the role of selected EE components in the reopening of visual cortex plasticity. In a first attempt to evaluate the efficacy of motor activity, social stimulation, or enhanced visual stimulation in promoting amblyopia recovery in the rat model, we reported a full recovery of ocular dominance and visual acuity both in animals experiencing high levels of voluntary motor activity in a running wheel and in rats exposed to a protocol of passive visual enrichment consisting on a rotating visual drum (Baroncelli et al., 2012), but not in animals subjected only to social enrichment. In agreement with previous results, those EE components found to be effective in triggering recovery from amblyopia were associated with a decreased GABA release in the visual cortex, without any change in the release of glutamate, thus resulting in a damped intracortical inhibition/excitation ratio (Baroncelli et al., 2012).

Among the various EE components, physical activity emerges as one of the most crucial, with numerous studies reporting its striking capability to mimic the more complex EE approach in producing a number of different beneficial effects (see Sale et al., 2014 for a recent survey). In a series of elegant works, Michael Stryker and colleagues have not only shown that locomotion powerfully increases visual responsiveness in the primary visual cortex (Niell and Stryker, 2010), but have also demonstrated that the enhancement of visual responses induced by locomotion is sufficient to promote recovery of visual function (Kaneko and Stryker, 2014) and provided evidence on the possible neural circuit underlying these effects (Fu et al., 2014, 2015; Lee et al., 2014). Niell and Stryker (2010) studied the response properties of neurons in primary visual cortex of awake mice while animals run on a freely rotating spherical treadmill with their heads fixed. They found that locomotor activity was associated with a dramatic increase in visual responsiveness in essentially all broad-spiking (presumed excitatory) cells without any concurrent changes in spontaneous firing rate or tuning properties; thus, increase in visually-evoked firing rate was not obtained at the expense of stimulus selectivity. The response magnitude of neurons in the visual thalamus was not affected by locomotion, indicating that the modulation of visual responses by locomotion is a cortical effect. Locomotion was also correlated with a decrease in low frequency power and an increase in the amplitude of the high-frequency gamma peak in the local EEG, suggesting a transition to a different cortical state during locomotion. Because the mice were not actively engaged in a perceptual task, and the increase in response was seen across the visual field, it seems likely that it reflects a general activation associated with locomotion, akin to arousal, rather than a mechanism of selective attention. The dependence on behavioral state was cell-type specific, in that a subset of the narrow-spiking cells (presumed inhibitory interneurons), which had little activity when the animal was stationary, began firing at high spontaneous rates during movement but then showed a suppressive response to visual stimuli.

It was evident that cortical activation associated with locomotion acted as a gain modulator, increasing responsiveness without changing selectivity. Thus, the increase in amplitude of visual responses during locomotion might provide a stronger drive for experience dependent visual cortical plasticity, increasing the capacity for recovery from early visual deprivation in the visual cortex of adult animals. In line with what we found in animals free to run on a running wheel in a condition of reversed suture (Baroncelli et al., 2012), visual responses to stimuli presented to the initially deprived eye during locomotion on a spherical treadmill (head fixed) increased significantly, reaching almost normal level after 7 days, and ocular dominance of visual cortical neurons was recovered (Kaneko and Stryker, 2014). The interesting thing is that increased responsiveness to deprived eye input and ocular dominance (OD) recovery was seen also under binocular vision, not only after reverse suture (reopening of the formerly deprived eye and closure of the non deprived eye). The recovery of response to stimuli presented to the formerly deprived eye was specific for the particular visual stimuli presented during locomotion, suggesting that recovery is facilitated only in the neural circuits that are activated during running.

Thus, it seems that enriching the environment in terms of voluntary motor activity and/or visual stimulation is a potentially useful strategy to reopen visual cortex plasticity and favor recovery of function in adult amblyopic subjects. How to apply the animal EE paradigm to humans is still debated. One approach very akin to EE is represented by active videogames, which combine various EE components such as visual attention and enhanced sensory stimulation (see Green and Bavelier, 2012). The videogame approach has been now tested in adult subjects with amblyopia, with encouraging results (Li et al., 2011).

Another promising strategy for amblyopia recovery which can be considered conceptually similar to EE is vPL, defined as the performance improvement, following practice, in visual tasks of different nature and complexity (see Bonaccorsi et al., 2014 for a recent review). A key property of vPL is its high specificity for the main stimulus attributes (e.g., stimulus orientation and location in the visual field), with the achieved performance typically returning to pre-learning baseline levels when test trials move to even slightly changed stimuli (McKee and Westheimer, 1978; Fiorentini and Berardi, 1980, 1981; Ball and Sekuler, 1982, 1987; Sale et al., 2011; see also Bonaccorsi et al., 2014).

vPL as a treatment for amblyopia has been first introduced in human patients, and it is currently considered one of the more promising strategies to favor recovery of visual functions in adult amblyopic subjects. The tasks used to elicit vPL are various, with examples of letter identification or longitudinal Vernier acuity and contrast sensitivity assessment (e.g., Levi and Polat, 1996; Levi et al., 1997; Polat et al., 2004; Li and Levi, 2004; Levi, 2005; Li et al., 2005, 2007; Chung et al., 2006, 2008; Zhou et al., 2006; Huang et al., 2008; Levi and Li, 2009). Significant improvements have been shown to be at reach in multiple domains, such as visual acuity, contrast sensitivity, and stereoacuity, with the learned improvements being frequently able to generalize to novel tasks (see Astle et al., 2011).

One consideration to make before moving to discuss the possible mechanisms of action of vPL and physical exercise in enhancing visual plasticity and promote recovery from amblyopia is that despite the interest for application of physical exercise and perceptual learning in the treatment of visual deficits in adult amblyopic subjects, to date there has been no attempt to understand whether the same procedures could also accelerate visual function recovery in developing subjects (both in humans and animal models). Such information might be instrumental for designing new therapeutic approaches aimed at speeding up the process of visual function recovery in amblyopic children.

## Neural Changes Underlying the Beneficial Effects of Physical Exercise and Visual Perceptual Learning on Visual Plasticity and Recovery from Amblyopia

### Physical Exercise

In the Niell and Stryker (2010) article, the authors made two predictions. First, if the narrow-spiking units whose visual response was suppressed during locomotion did turn out to be inhibitory, they might play a crucial role in the cortical response to locomotor activation. Their increased firing rate during locomotion would increase overall inhibition, serving to keep spontaneous rates relatively constant; in the presence of a visual stimulus, the reduction in their firing would relieve this inhibition, allowing the high-amplitude responses observed during locomotion.

Second, they hypothesized a role for the neuromodulator acetylcholine (Ach). ACh has been demonstrated to play a role in cortical activation and attentional modulation in many systems (Hasselmo and Giocomo, 2006; Weinberger, 2007). The shift from low to high frequency in the local EEG spectrum is a characteristic of the actions of Ach and in particular of nucleus basalis stimulation (Buzsaki et al., 1988; Metherate et al., 1992; Rodriguez et al., 2004). Furthermore, cholinergic agonists have been shown to enhance visual responses in V1, without significant change in selectivity or spontaneous rate (Sillito and Kemp, 1983; Sato et al., 1987). In addition, nucleus basalis stimulation in the anesthetized rat has been shown to increase the reliability of visual responses to movies of natural scenes (Goard and Dan, 2009).

To dissect the circuits underlying locomotion effects on visual cortical responsiveness, Fu et al. (2014) took advantage of advances in mouse genetics and *in vivo* imaging technology to characterize the responses of different types of inhibitory neurons in mouse V1 in awake animals free to run on the spherical treadmill. In mice with Vasoactive Intestinal Peptide (VIP)-positive GABAergic neurons genetically labelled, the authors imaged the calcium responses of these VIP neurons in freely running head-fixed mice with or without visual stimulation. They found that the neural activity of VIP neurons, but not of non VIP neurons, is greatly elevated during locomotion even without visual stimulation. Visual stimulation, which drove the other cortical neurons, did not further increase the activation of VIP neurons by locomotion. A similar approach revealed that somatostatin (SST) neurons were inhibited by locomotion, consistent with a circuit in which VIP cells increase activity of neighboring excitatory cells by inhibiting their inhibitory input from SST cells. Activating VIP neurons in mouse V1 by means of optogenetic in stationary mice mimicked the effect of locomotion and increased the visual responses of visual cortical neurons, while focal damage to VIP neurons blocked the enhancement of cortical responses by locomotion.

The local blockade of nicotinic cholinergic input, but not of glutamatergic input, reduced the response of VIP neurons to locomotion by more than two thirds, and measurements *in vitro* disclosed powerful nicotinic cholinergic input to VIP neurons. Consistent with this result, upper layer VIP neurons in V1 turned out to receive direct input from the nucleus of the diagonal band of Broca (NDB), a cholinergic center in basal forebrain (Fu et al., 2014).

The cortical VIP-SOM circuit is also the mediator of locomotion induced enhancement of adult visual cortical plasticity (Fu et al., 2015). Genetically silencing VIP synaptic transmission in binocular zone of mouse V1 prevents locomotion from enhancing recovery of the amblyopic eye cortical responses. The involvement of VIP neurons in locomotion-induced enhancement of adult V1 plasticity has also been shown with a different approach, that is employing a brief MD in adult mice as a probe for OD plasticity. Between four to five days MD is insufficient to significantly shift OD in adult mice, due to the low OD plasticity, but they become effective if coupled with locomotion. Genetically silencing VIP neuron synaptic transmission in running mice makes MD ineffective in shifting OD of cortical neurons; optogenetic activation of VIP neurons in non running mice reproduced the plasticity-enhancement effects of locomotion. Silencing SST neurons turned out to be as effective as activating VIP neurons for enhancing adult OD plasticity in response to brief MD.

Altogether, these results are consistent with the idea that reduced inhibition is permissive for enhancing adult visual cortical plasticity (Harauzov et al., 2010; Sale et al., 2014) and reveal a disinhibitory circuit, VIP-SOM, that may underlie the reduction in GABA content and release in V1 found in EE or physical exercised mice in correlation with a strong enhancement of V1 plasticity and with recovery from amblyopia (Sale et al., 2007; Baroncelli et al., 2010, 2012).

The starting point of this cholinergic-VIP-SOM neuron mediated cortical response enhancement seems to be the mesencephalic locomotor region (MLR; Lee et al., 2014). MLR is the midbrain region the activation of which is sufficient to induce locomotion and is associated with the “ascending reticular activating system” described by Moruzzi and Magoun; electrical stimulation of this region can induce physiological correlates of alertness, such as desynchronization of low-frequency oscillations (<10 Hz) of the electroencephalogram (Moruzzi and Magoun, 1949).

Lee et al. (2014) found that optogenetic stimulation of MLR in awake, head-fixed mice induced both locomotion and increases in the gain of cortical responses in V1. Subthreshold optogenetic stimulation of the MLR was sufficient to increase the gain of visual responses and enhance gamma oscillations similar to those normally associated with locomotion even in the absence of overt movement. Furthermore, stimulation of axon terminals projecting from the MLR to cholinergic basal forebrain also reproduced this effect, suggesting that the MLR can influence cortical processing, potentially through projections directed toward the basal forebrain. These findings can be used to build a simple model in which the MLR initiates locomotion through descending pathways to the spinal cord while coordinating changes in brain state through its ascending projections.

Running and locomotion is associated not only with activation of cortical VIP neurons, but also with increases in multiple neuromodulators, including serotonin, which has been shown to be enhanced in V1 by EE (Baroncelli et al., 2010) and to promote adult V1 plasticity (Maya Vetencourt et al., 2008); interestingly, VIP neurons express the 5-HT3 serotonin receptor (Lee et al., 2014), suggesting that the VIP-SST disinhibitory circuit might be involved also in the effects of 5-HT on cortical plasticity. However, as suggested by Fu et al. (2015), enhancement of adult plasticity by locomotion is likely to be more complex than simply activating VIP-SST disinhibitory circuit.

### Visual Perceptual Learning

vPL has been related to a number of different cellular mechanisms, including an increase in the number of neurons representing the learned stimulus (Recanzone et al., 1992, 1993) or, alternatively, more subtle functional changes at the levels of single neurons, both in terms of response strength and tuning (see Schummers et al., 2005). An obvious candidate mechanism underlying the cortical changes induced by vPL is synaptic plasticity. Gilbert and Li (2013) proposed that vPL is associated with long-term changes in either top-down circuits conveying information about attention and behavioral expectation, and in bottom-up circuits directly involved in experience-dependent changes. In agreement with this model, neurons in V1 are known to be capable of extensive integration well beyond the borders of their receptive fields, a property dependent of the existence of strong horizontal connections linking columns with similar orientation preference (Stettler et al., 2002; Stepanyants et al., 2009). While changes in favor of synaptic plasticity processes underlying perceptual learning has been recorded both in the primary motor cortex and in V1 in humans and non human primates (e.g., Rioult-Pedotti et al., 2000; Li et al., 2008; Yotsumoto et al., 2008), conclusive evidence has remained elusive.

The vPL approach in humans has informed and inspired increasing experimental work in simple animal models, mostly devoted to understand the mechanisms underlying the remarkable effects elicited by visual training. Frenkel et al. (2006) reported that, in the mouse, exposure to visual stimuli represented by gratings of a given orientation potentiates visual responses to the same orientation, an NMDA dependent experience-induced response enhancement called stimulus-selective response potentiation (SRP). We recently reported a more direct evidence for a synaptic plasticity engagement in vPL (Sale et al., 2011). To elicit vPL, we trained adult rats in a visual discrimination task in which they had to discriminate two visual gratings of very different spatial frequency; once the animals learned the task, the two stimuli were rendered progressively more similar to each other, providing a training process with increasing task difficulty. We observed a progressive improvement of discrimination ability with training, with performance reaching a steady plateau after few days. This kind of vPL turned out to be strictly selective for the orientation of the gratings employed during training, indicating that it requires activation of V1 circuits. A group of control rats learned the associative task in which they were required to discriminate two gratings of very different spatial frequency and then practiced with this easily discriminable pair of gratings for the same amount of days as vPL animals. When tested within 1 h from the last discrimination trial, long-term potentiation (LTP) elicited by theta burst stimulation applied to layer II-III of V1 slices appeared occluded in vPL animals compared to controls, both when testing its inducibility in vertical and horizontal connections. Moreover, the amplitudes of field potentials turned out to be increased in trained animals compared to controls, indicating that learning leads to a synaptic potentiation of V1 connections; no potentiation or LTP occlusion was found in control animals (Sale et al., 2011). These data provide a strong indication that the improvements displayed by vPL rats can be explained in terms of long-term increments of synaptic efficacy in V1 caused by learning, as already well known for different brain regions, such as hippocampus, amygdala and motor cortex (Rogan et al., 1997; Rioult-Pedotti et al., 1998; Whitlock et al., 2006).

The possibility to strengthen synaptic efficacy using vPL is attractive in terms of application to amblyopia therapy. Adult amblyopic rats trained in the same vPL task displayed robust recovery of visual acuity and ocular dominance (Baroncelli et al., 2012), an effect persisting for quite a long time (i.e., 14 days, corresponding to at least 20 months in the timescale of human life). In search for possible molecular candidates underlying the beneficial effects of vPL, we found that it resulted in a decrease of the inhibition-excitation balance in V1. Vision recovery was instead totally absent in those control groups in which the treatment did not induce LTP in V1, i.e., in rats that were trained only until the first step of the discrimination procedure between the test and the reference grating, without proceeding further with a progression of finer discrimination trials (Baroncelli et al., 2012). The control group performed an equal amount of physical activity in the maze with respect to the animals trained in the vPL task, ruling out the possibility that the physical exercise component intrinsic to the employed vPL procedure might contribute to visual function recovery. This conclusion could seem at odd with the striking capability of running (Baroncelli et al., 2012) to promote recovery of ocular dominance and visual acuity in amblyopic subjects. It has to be noted, however, that while, in the study by Baroncelli et al. (2012) running was a form of voluntary exercise, swimming activity in the water maze is necessarily imposed. A vast literature exists pointing out different effects elicited by voluntary vs. forced motor behavior on brain and behavior, in terms of activated monoamine neurotransmitters (Dishman et al., 1997), hippocampal parvalbumin expression (Arida et al., 2004), hippocampal BDNF and synapsin-1 expression (Ploughman et al., 2005), longevity and body composition (Narath et al., 2001), taste aversion learning (Masaki and Nakajima, 2006) and open-field behavior (Burghardt et al., 2004).

## Concluding Remarks

The research reviewed here has demonstrated that voluntary physical exercise and vPL, two totally non invasive procedures, share the remarkable capability to potentiate plasticity in the adult visual cortex, favoring recovering of visual functions in adult amblyopic rodents.

These procedures have a great potential for application to human subjects, and indeed vPL has been first introduced in clinical research and then modeled in rodents. On the contrary, the impact of physical exercise on amblyopic adults remains to be elucidated, with preliminary results in our laboratory showing a strong enhancement of visual cortical plasticity in healthy subjects after a period of voluntary physical activity (Lunghi and Sale, in press).

In parallel with experiments aimed at directly testing the effects of the proposed behavioral interventions in human patients, a number of still open questions should be addressed in the animal model to strengthen the transferability of the achieved results:

- Is it possible to induce recovery of visual functional in animals trained with vPL without performing reverse suture, i.e., in adult amblyopic subjects with both eyes open?
- Which is the impact of physical exercise or vPL on stereopsis abilities in amblyopic animals?
- Which is the role of selected classes of inhibitory interneurons in the beneficial effects elicited by physical or visual training?
- Are the effects of visual recovery persistent?
- Which are the molecular mechanisms underlying plasticity in exercised animals?

Future research should focus on these open issues.

## Conflict of Interest Statement

The authors declare that the research was conducted in the absence of any commercial or financial relationships that could be construed as a potential conflict of interest.

## References

[B1] Antonini A. Stryker M. P. (1998). Effect of sensory disuse on geniculate afferents to cat visual cortex. Vis. Neurosci. 15, 401–409. 10.1017/s0952523898153105 9685193 PMC2430473

[B2] Arida R. M. Scorza C. A. da Silva A. V. Scorza F. A. Cavalheiro E. A. (2004). Differential effects of spontaneous versus forced exercise in rats on the staining of parvalbumin-positive neurons in the hippocampal formation. Neurosci. Lett. 364, 135–138. 10.1016/j.neulet.2004.03.086 15196661

[B3] Astle A. T. McGraw P. V. Webb B. S. (2011). Can human amblyopia be treated in adulthood?. Strabismus 19, 99–109. 10.3109/09273972.2011.600420 21870913 PMC3433677

[B4] Ball K. Sekuler R. (1982). A specific and enduring improvement in visual motion discrimination. Science 218, 697–698. 10.1126/science.7134968 7134968

[B5] Ball K. Sekuler R. (1987). Direction-specific improvement in motion discrimination. Vision Res. 27, 953–965. 10.1016/0042-6989(87)90011-3 3660656

[B7] Bao S. Chan V. T. Merzenich M. M. (2001). Cortical remodelling induced by activity of ventral tegmental dopamine neurons. Nature 412, 79–83. 10.1038/35083586 11452310

[B8] Baro J. A. Lehmkuhle S. Kratz K. E. (1990). Electroretinograms and visual evoked potentials in long-term monocularly deprived cats. Invest. Ophthalmol. Vis. Sci. 31, 1405–1409. 2365572

[B9] Baroncelli L. Bonaccorsi J. Milanese M. Bonifacino T. Giribaldi F. Manno I.. (2012). Enriched experience and recovery from amblyopia in adult rats: impact of motor, social and sensory components. Neuropharmacology 62, 2388–2397. 10.1016/j.neuropharm.2012.02.010 22532989

[B10] Baroncelli L. Braschi C. Maffei L. (2013). Visual depth perception in normal and deprived rats: effects of environmental enrichment. Neuroscience 236, 313–319. 10.1016/j.neuroscience.2013.01.036 23357122

[B11] Baroncelli L. Maffei L. Sale A. (2011). New perspectives in amblyopia therapy on adults: a critical role for the excitatory/inhibitory balance. Front. Cell. Neurosci. 5:25. 10.3389/fncel.2011.00025 22144947 PMC3223381

[B12] Baroncelli L. Sale A. Viegi A. Maya Vetencourt J. F. De Pasquale R. Baldini S.. (2010). Experience-dependent reactivation of ocular dominance plasticity in the adult visual cortex. Exp. Neurol. 226, 100–109. 10.1016/j.expneurol.2010.08.009 20713044

[B13] Barrett B. T. Bradley A. McGraw P. V. (2004). Understanding the neural basis of amblyopia. Neuroscientist 10, 106–117. 10.1177/1073858403262153 15070485

[B14] Bavelier D. Levi D. M. Li R. W. Dan Y. Hensch T. K. (2010). Removing brakes on adult brain plasticity: from molecular to behavioral interventions. J. Neurosci. 30, 14964–14971. 10.1523/JNEUROSCI.4812-10.2010 21068299 PMC2992973

[B15] Bear M. F. Singer W. (1986). Modulation of visual cortical plasticity by acetylcholine and noradrenaline. Nature 320, 172–176. 10.1038/320172a0 3005879

[B17] Beurdeley M. Spatazza J. Lee H. H. Sugiyama S. Bernard C. Di Nardo A. A.. (2012). Otx2 binding to perineuronal nets persistently regulates plasticity in the mature visual cortex. J. Neurosci. 32, 9429–9437. 10.1523/JNEUROSCI.0394-12.2012 22764251 PMC3419577

[B18] Blakemore C. Vital-Durand F. Garey L. J. (1981). Recovery from monocular deprivation in the monkey. I. Reversal of physiological effects in the visual cortex. Proc. R. Soc. Lond. B Biol. Sci. 213, 399–423. 10.1098/rspb.1981.0072 6119688

[B104] Bonaccorsi J. Berardi N. Sale A. (2014). Treatment of amblyopia in the adult: insights from a new rodent model of visual perceptual learning. Front. Neural Circuits 8:82. 10.3389/fncir.2014.00082 25076874 PMC4100600

[B19] Burghardt P. R. Fulk L. J. Hand G. A. Wilson M. A. (2004). The effects of chronic treadmill and wheel running on behavior in rats. Brain Res. 1019, 84–96. 10.1016/j.brainres.2004.05.086 15306242

[B20] Buzsaki G. Bickford R. R. Ponomareff G. Thal L. L. Mandel R. Gage F. F. (1988). Nucleus basalis and thalamic control of neocortical activity in the freely moving rat. J. Neurosci. 8, 4007–4026. 3183710 10.1523/JNEUROSCI.08-11-04007.1988 PMC6569493

[B21] Chung S. T. Li R. W. Levi D. M. (2006). Identification of contrast-defined letters benefits from perceptual learning in adults with amblyopia. Vision Res. 46, 3853–3861. 10.1016/j.visres.2006.06.014 16930666 PMC1852540

[B22] Chung S. T. Li R. W. Levi D. M. (2008). Learning to identify near-threshold luminance-defined and contrast-defined letters in observers with amblyopia. Vision Res. 48, 2739–2750. 10.1016/j.visres.2008.09.009 18824189 PMC2642955

[B24] Dishman R. K. Renner K. J. Youngstedt S. D. Reigle T. G. Bunnell B. N. Burke K. A.. (1997). Activity wheel running reduces escape latency and alters brain monoamine levels after footshock. Brain Res. Bull. 42, 399–406. 10.1016/s0361-9230(96)00329-2 9092882

[B26] Fiorentini A. Berardi N. (1980). Perceptual learning specific for orientation and spatial frequency. Nature 287, 43–44. 10.1038/287043a0 7412873

[B27] Fiorentini A. Berardi N. (1981). Learning in grating waveform discrimination: specificity for orientation and spatial frequency. Vision Res. 21, 1149–1158. 10.1016/0042-6989(81)90017-1 7314493

[B28] Foley-Nolan A. McCann A. O’Keefe M. (1997). Atropine penalisation versus occlusion as the primary treatment for amblyopia. Br. J. Ophthalmol. 81, 54–57. 10.1136/bjo.81.1.54 9135409 PMC1721990

[B29] Frenkel M. Y. Sawtell N. B. Diogo A. C. Yoon B. Neve R. L. Bear M. F. (2006). Instructive effect of visual experience in mouse visual cortex. Neuron 51, 339–349. 10.1016/j.neuron.2006.06.026 16880128

[B31] Fu Y. Kaneko M. Tang Y. Alvarez-Buylla A. Stryker M. M. (2015). A cortical disinhibitory circuit for enhancing adult plasticity. Elife 4:e05558. 10.7554/elife.05558 25626167 PMC4337686

[B30] Fu Y. Tucciarone J. J. Espinosa J. J. Sheng N. Darcy D. D. Nicoll R. R.. (2014). A cortical circuit for gain control by behavioral state. Cell 13, 1139–1152. 10.1016/j.cell.2014.01.050 24630718 PMC4041382

[B32] Gilbert C. D. Li W. (2013). Top-down influences on visual processing. Nat. Rev. Neurosci. 14, 350–363. 10.1038/nrn3476 23595013 PMC3864796

[B33] Goard M. Dan Y. (2009). Basal forebrain activation enhances cortical coding of natural scenes. Nat. Neurosci. 12, 1444–1449. 10.1038/nn.2402 19801988 PMC3576925

[B35] Green C. C. Bavelier D. (2012). Learning, attentional control and action video games. Curr. Biol. 22, R197–R206. 10.1016/j.cub.2012.02.012 22440805 PMC3461277

[B36] Harauzov A. Spolidoro M. DiCristo G. De Pasquale R. Cancedda L. Pizzorusso T.. (2010). Reducing intracortical inhibition in the adult visual cortex promotes ocular dominance plasticity. J. Neurosci. 30, 361–371. 10.1523/JNEUROSCI.2233-09.2010 20053917 PMC6632513

[B37] Hasselmo M. M. Giocomo L. L. (2006). Cholinergic modulation of cortical function. J. Mol. Neurosci. 30, 133–135. 10.1385/JMN:30:1:133 17192659

[B38] He H. Y. Ray B. Dennis K. Quinlan E. M. (2007). Experience-dependent recovery of vision following chronic deprivation amblyopia. Nat. Neurosci. 10, 1134–1136. 10.1038/nn1965 17694050

[B39] Hensch T. K. (2005). Critical period plasticity in local cortical circuits. Nat. Rev. Neurosci. 6, 877–888. 10.1038/nrn1787 16261181

[B40] Hess R. F. (2001). Amblyopia: site unseen. Clin. Exp. Optom. 84, 321–336. 10.1111/j.1444-0938.2001.tb06604.x 12366358

[B41] Holmes J. M. Clarke M. P. (2006). Amblyopia. Lancet 367, 1343–1351. 10.1016/S0140-6736(06)68581-4 16631913

[B42] Huang C. B. Zhou Y. Lu Z. L. (2008). Broad bandwidth of perceptual learning in the visual system of adults with anisometropic amblyopia. Proc. Natl. Acad. Sci. U S A 105, 4068–4073. 10.1073/pnas.0800824105 18316716 PMC2268837

[B43] Hubel D. H. Wiesel T. N. (1970). The period of susceptibility to the physiological effects of unilateral eye closure in kittens. J. Physiol. 206, 419–436. 10.1113/jphysiol.1970.sp009022 5498493 PMC1348655

[B44] Kaneko M. Stryker M. M. (2014). Sensory experience during locomotion promotes recovery of function in adult visual cortex. Elife 3:e02798. 10.7554/elife.02798 24970838 PMC4070284

[B45] Kasamatsu T. Pettigrew J. D. (1976). Depletion of brain catecholamines: failure of ocular dominance shift after monocular occlusion in kittens. Science 194, 206–209. 10.1126/science.959850 959850

[B46] Kilgard M. P. Merzenich M. M. (1998). Cortical map reorganization enabled by nucleus basalis activity. Science 279, 1714–1718. 10.1126/science.279.5357.1714 9497289

[B47] Kratz K. E. Mangel S. C. Lehmkuhle S. Sherman M. (1979). Retinal X- and Y-cells in monocularly lid-sutured cats: normality of spatial and temporal properties. Brain Res. 172, 545–551. 10.1016/0006-8993(79)90586-9 476497

[B48] Lee A. A. Hoy J. J. Bonci A. Wilbrecht L. Stryker M. M. Niell C. C. (2014). Identification of a brainstem circuit regulating visual cortical state in parallel with locomotion. Neuron 83, 455–466. 10.1016/j.neuron.2014.06.031 25033185 PMC4151326

[B49] Levi D. M. (2005). Perceptual learning in adults with amblyopia: a reevaluation of critical periods in human vision. Dev. Psychobiol. 46, 222–232. 10.1002/dev.20050 15772964

[B52] Levi D. D. Li R. W. (2009). Perceptual learning as a potential treatment for amblyopia: a mini-review. Vision Res. 49, 2535–2549. 10.1016/j.visres.2009.02.010 19250947 PMC2764839

[B50] Levi D. M. Polat U. (1996). Neural plasticity in adults with amblyopia. Proc. Natl. Acad. Sci. U S A 93, 6830–6834. 10.1073/pnas.93.13.6830 8692904 PMC39113

[B51] Levi D. M. Polat U. Hu Y. S. (1997). Improvement in Vernier acuity in adults with amblyopia. Practice makes better. Invest. Ophthalmol. Vis. Sci. 38, 1493–1510. 9224277

[B53] Lewis T. L. Maurer D. (2009). Effects of early pattern deprivation on visual development. Optom. Vis. Sci. 86, 640–646. 10.1097/opx.0b013e3181a7296b 19417706

[B250] Li R. W. Levi D. M. (2004). Characterizing the mechanisms of improvement for position discrimination in adult amblyopia. J. Vis. 4, 476–487. 15330715 10.1167/4.6.7

[B54] Li R. R. Ngo C. Nguyen J. Levi D. D. (2011). Video-game play induces plasticity in the visual system of adults with amblyopia. PLoS Biol. 9:e1001135. 10.1371/journal.pbio.1001135 21912514 PMC3166159

[B55] Li R. R. Provost A. Levi D. D. (2007). Extended perceptual learning results in substantial recovery of positional acuity and visual acuity in juvenile amblyopia. Invest. Ophthalmol. Vis. Sci. 48, 5046–5051. 10.1167/iovs.07-0324 17962456

[B56] Li R. R. Young K. K. Hoenig P. Levi D. D. (2005). Perceptual learning improves visual performance in juvenile amblyopia. Invest. Ophthalmol. Vis. Sci. 46, 3161–3168. 10.1167/iovs.05-0286 16123415

[B57] Li W. Piech V. Gilbert C. C. (2008). Learning to link visual contours. Neuron 57, 442–451. 10.1016/j.neuron.2007.12.011 18255036 PMC2409109

[B105] Lunghi C. Sale A. (in press). A cycling lane for brain rewiring. Curr. Biol. 10.1016/j.cub.2015.10.026 PMC5040496 26654367

[B58] Masaki T. Nakajima S. (2006). Taste aversion in rats induced by forced swimming, voluntary running, forced running and lithium chloride injection treatments. Physiol. Behav. 88, 411–416. 10.1016/j.physbeh.2006.04.013 16777150

[B59] Maya Vetencourt J. F. Sale A. Viegi A. Baroncelli L. De Pasquale R. O’Leary O. F.. (2008). The antidepressant fluoxetine restores plasticity in the adult visual cortex. Science 320, 385–388. 10.1126/science.1150516 18420937

[B60] McKee S. P. Westheimer G. (1978). Improvement in vernier acuity with practice. Percept. Psychophys. 24, 258–262. 10.3758/bf03206097 704286

[B61] Metherate R. Cox C. C. Ashe J. J. (1992). Cellular bases of neocortical activation: modulation of neural oscillations by the nucleus basalis and endogenous acetylcholine. J. Neurosci. 12, 4701–4711. 1361197 10.1523/JNEUROSCI.12-12-04701.1992 PMC6575759

[B62] Montey K. K. Quinlan E. E. (2011). Recovery from chronic monocular deprivation following reactivation of thalamocortical plasticity by dark exposure. Nat. Commun. 2:317. 10.1038/ncomms1312 21587234 PMC4620663

[B63] Morishita H. Miwa J. M. Heintz N. Hensch T. K. (2010). Lynx1, a cholinergic brake, limits plasticity in adult visual cortex. Science 330, 1238–1240. 10.1126/science.1195320 21071629 PMC3387538

[B64] Moruzzi G. Magoun H. H. (1949). Brain stem reticular formation and activation of the EEG. Electroencephalogr. Clin. Neurophysiol. 1, 455–473. 10.1016/0013-4694(49)90219-9 18421835

[B65] Movshon J. A. Dürsteler M. R. (1977). Effects of brief periods of unilateral eye closure on the kitten’s visual system. J. Neurophysiol. 40, 1255–1265. 925727 10.1152/jn.1977.40.6.1255

[B66] Narath E. Skalicky M. Viidik A. (2001). Voluntary and forced exercise influence the survival and body composition of ageing male rats differently. Exp. Gerontol. 36, 1699–1711. 10.1016/s0531-5565(01)00145-0 11672990

[B67] Niell C. C. Stryker M. M. (2010). Modulation of visual responses by behavioral state in mouse visual cortex. Neuron 65, 472–479. 10.1016/j.neuron.2010.01.033 20188652 PMC3184003

[B68] Olson C. R. Freeman R. D. (1975). Progressive changes in kitten striate cortex during monocular vision. J. Neurophysiol. 38, 26–32. 162944 10.1152/jn.1975.38.1.26

[B69] Pediatric Eye Disease Investigator Group (2002). A randomized trial of atropine vs. patching for treatment of moderate amblyopia in children. Arch. Ophthalmol. 120, 268–278. 10.1001/archopht.120.3.268 11879129

[B70] Pizzorusso T. Medini P. Berardi N. Chierzi S. Fawcett J. J. Maffei L. (2002). Reactivation of ocular dominance plasticity in the adult visual cortex. Science 298, 1248–1251. 10.1126/science.1072699 12424383

[B71] Pizzorusso T. Medini P. Landi S. Baldini S. Berardi N. Maffei L. (2006). Structural and functional recovery from early monocular deprivation in adult rats. Proc. Natl. Acad. Sci. U S A 103, 8517–8522. 10.1073/pnas.0602657103 16709670 PMC1482523

[B72] Ploughman M. Granter-Button S. Chernenko G. Tucker B. A. Mearow K. M. Corbett D. (2005). Endurance exercise regimens induce differential effects on brain-derived neurotrophic factor, synapsin-I and insulin-like growth factor I after focal ischemia. Neuroscience 136, 991–1001. 10.1016/j.neuroscience.2005.08.037 16203102

[B73] Polat U. Ma-Naim T. Belkin M. Sagi D. (2004). Improving vision in adult amblyopia by perceptual learning. Proc. Natl. Acad. Sci. U S A 101, 6692–6697. 10.1073/pnas.0401200101 15096608 PMC404107

[B74] Putignano E. Lonetti G. Cancedda L. Ratto G. Costa M. Maffei L.. (2007). Developmental downregulation of histone posttranslational modifications regulates visual cortical plasticity. Neuron 53, 747–759. 10.1016/j.neuron.2007.02.007 17329213

[B75] Recanzone G. H. Jenkins W. M. Hradek G. T. Merzenich M. M. (1992). Progressive improvement in discriminative abilities in adult owl monkeys performing a tactile frequency discrimination task. J. Neurophysiol. 67, 1015–1030. 1597695 10.1152/jn.1992.67.5.1015

[B76] Recanzone G. H. Schreiner C. E. Merzenich M. M. (1993). Plasticity in the frequency representation of primary auditory cortex following discrimination training in adult owl monkeys. J. Neurosci. 13, 87–103. 8423485 10.1523/JNEUROSCI.13-01-00087.1993 PMC6576321

[B77] Rioult-Pedotti M. S. Friedman D. Donoghue J. P. (2000). Learning-induced LTP in neocortex. Science 290, 533–536. 10.1126/science.290.5491.533 11039938

[B78] Rioult-Pedotti M. S. Friedman D. Hess G. Donoghue J. P. (1998). Strengthening of horizontal cortical connections following skill learning. Nat. Neurosci. 1, 230–234. 10.1038/678 10195148

[B79] Rodriguez R. Kallenbach U. Singer W. Munk M. M. (2004). Short- and long-term effects of cholinergic modulation on gamma oscillations and response synchronization in the visual cortex. J. Neurosci. 24, 10369–10378. 10.1523/jneurosci.1839-04.2004 15548651 PMC6730306

[B80] Rogan M. T. Staubli U. V. LeDoux J. E. (1997). Fear conditioning induces associative long-term potentiation in the amygdala. Nature 390, 604–607. 9403688 10.1038/37601

[B81] Sale A. Berardi N. Maffei L. (2014). Environment and brain plasticity: towards an endogenous pharmacotherapy. Physiol. Rev. 94, 189–234. 10.1152/physrev.00036.2012 24382886

[B82] Sale A. De Pasquale R. Bonaccorsi J. Pietra G. Olivieri D. Berardi N.. (2011). Visual perceptual learning induces long-term potentiation in the visual cortex. Neuroscience 172, 219–225. 10.1016/j.neuroscience.2010.10.078 21056088

[B83] Sale A. Maya Vetencourt J. F. Medini P. Cenni M. C. Baroncelli L. De Pasquale R.. (2007). Environmental enrichment in adulthood promotes amblyopia recovery through a reduction of intracortical inhibition. Nat. Neurosci. 10, 679–681. 10.1038/nn1899 17468749

[B84] Sato H. Hata Y. Masui H. Tsumoto T. (1987). A functional role of cholinergic innervation to neurons in the cat visual cortex. J. Neurophysiol. 58, 765–780. 3681394 10.1152/jn.1987.58.4.765

[B85] Scali M. Baroncelli L. Cenni M. M. Sale A. Maffei L. (2012). A rich environmental experience reactivates visual cortex plasticity in aged rats. Exp. Gerontol. 47, 337–341. 10.1016/j.exger.2012.01.007 22329907

[B86] Schummers J. Sharma J. Sur M. (2005). Bottom-up and top-down dynamics in visual cortex. Prog. Brain Res. 149, 65–81. 10.1016/s0079-6123(05)49006-8 16226577

[B87] Sherman S. M. Stone J. (1973). Physiological normality of the retinal in visually deprived cats. Brain Res. 60, 224–230. 10.1016/0006-8993(73)90861-5 4744763

[B88] Silingardi D. Scali M. Belluomini G. Pizzorusso T. (2010). Epigenetic treatments of adult rats promote recovery from visual acuity deficits induced by long-term monocular deprivation. Eur. J. Neurosci. 31, 2185–2192. 10.1111/j.1460-9568.2010.07261.x 20550570

[B89] Sillito A. A. Kemp J. J. (1983). Cholinergic modulation of the functional organization of the cat visual cortex. Brain Res. 289, 143–155. 10.1016/0006-8993(83)90015-x 6661640

[B90] Simons K. Stein L. Sener E. C. Vitale S. Guyton D. L. (1997). Full-time atropine, intermittent atropine and optical penalization and binocular outcome in treatment of strabismic amblyopia. Ophthalmology 104, 2143–2155. 10.1016/s0161-6420(97)30048-7 9400777

[B91] Southwell D. G. Froemke R. C. Alvarez-Buylla A. Stryker M. P. Gandhi S. S. (2010). Cortical plasticity induced by inhibitory neuron transplantation. Science 327, 1145–1148. 10.1126/science.1183962 20185728 PMC3164148

[B92] Stepanyants A. Martinez L. M. Ferecskó A. S. Kisvárday Z. F. (2009). The fractions of short- and long-range connections in the visual cortex. Proc. Natl. Acad. Sci. U S A 106, 3555–3560. 10.1073/pnas.0810390106 19221032 PMC2651285

[B93] Stettler D. D. Das A. Bennett J. Gilbert C. D. (2002). Lateral connectivity and contextual interactions in macaque primary visual cortex. Neuron 36, 739–750. 10.1016/s0896-6273(02)01029-2 12441061

[B94] Sugiyama S. Di Nardo A. A. Aizawa S. Matsuo I. Volovitch M. Prochiantz A.. (2008). Experience-dependent transfer of Otx2 homeoprotein into the visual cortex activates postnatal plasticity. Cell 134, 508–520. 10.1016/j.cell.2008.05.054 18692473

[B95] van Praag H. Kempermann G. Gage F. H. (2000). Neural consequences of environmental enrichment. Nat. Rev. Neurosci. 1, 191–198. 10.1038/35042057 11257907

[B97] Weinberger N. N. (2007). Associative representational plasticity in the auditory cortex: a synthesis of two disciplines. Learn. Mem. 14, 1–16. 10.1101/lm.421807 17202426 PMC3601844

[B98] Whitlock J. R. Heynen A. J. Shuler M. G. Bear M. F. (2006). Learning induces long-term potentiation in the hippocampus. Science 313, 1093–1097. 10.1126/science.1128134 16931756

[B99] Wiesel T. N. Hubel D. H. (1963). Single-cell responses in striate cortex of kittens deprived of vision in one eye. J. Neurophysiol. 26, 1003–1017. 14084161 10.1152/jn.1963.26.6.1003

[B100] Wu C. Hunter D. G. (2006). Amblyopia: diagnostic and therapeutic options. Am. J. Ophthalmol. 141, 175–184. 10.1016/j.ajo.2005.07.060 16386994

[B101] Yotsumoto Y. Watanabe T. Sasaki Y. (2008). Different dynamics of performance and brain activation in the time course of perceptual learning. Neuron 57, 827–833. 10.1016/j.neuron.2008.02.034 18367084 PMC2735208

[B102] Zhang T. Y. Meaney M. J. (2010). Epigenetics and the environmental regulation of the genome and its function. Annu. Rev. Psychol. 61, 439–466, C431–433. 10.1146/annurev.psych.60.110707.163625 19958180

[B103] Zhou Y. Huang C. Xu P. Tao L. Qiu Z. Li X.. (2006). Perceptual learning improves contrast sensitivity and visual acuity in adults with anisometropic amblyopia. Vision Res. 46, 739–750. 10.1016/j.visres.2005.07.031 16153674

